# *N*-(Diphenyl­vinyl­idene)-2,6-diisopropyl­aniline

**DOI:** 10.1107/S1600536808040397

**Published:** 2008-12-06

**Authors:** Wolfgang Imhof

**Affiliations:** a Institute of Inorganic and Analytical Chemistry, Friedrich Schiller University, August-Bebel-Strasse 2, 07743 Jena, Germany

## Abstract

The title compound, C_26_H_27_N, was prepared by the elimination of water from *N*-(2,6-diisopropyl­phen­yl)-2,2-diphenyl­acetamide. The angle at the central C atom of the cumulene measures 172.5 (4)°. Mol­ecules are connected into infinite chains by inter­molecular C—H⋯N inter­actions.

## Related literature

For the synthetic procedure, see: Stevens & Singhal (1964 ▶). For related structures, see: Naqvi & Wheatley (1970 ▶); Jochims *et al.* (1984 ▶); Kuipers *et al.* (1989 ▶). For general background, see: Imhof (1997*a* ▶,*b* ▶). For properties of weak hydrogen bonds, see: Desiraju & Steiner (1999 ▶).
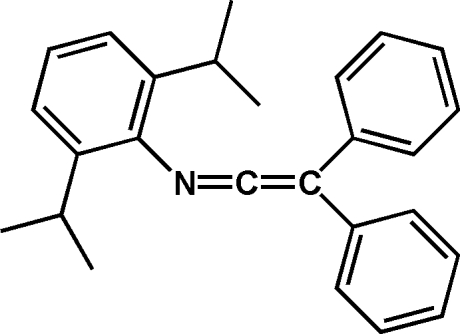


## Experimental

### 

#### Crystal data

C_26_H_27_N
*M* *_r_* = 353.49
Orthorhombic, 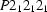

*a* = 8.082 (4) Å
*b* = 14.308 (4) Å
*c* = 17.790 (2) Å
*V* = 2057 (1) Å^3^
*Z* = 4
Mo *K*α radiation
μ = 0.07 mm^−1^
*T* = 173 (2) K
0.3 × 0.2 × 0.2 mm

#### Data collection

Enraf–Nonius CAD-4 diffractometer
Absorption correction: none
3554 measured reflections
1853 independent reflections
1531 reflections with *I* > 2σ(*I*)
*R*_int_ = 0.065
θ_max_ = 24.0°
3 standard reflections frequency: 120 min intensity decay: <0.1%

#### Refinement

*R*[*F* ^2^ > 2σ(*F* ^2^)] = 0.040
*wR*(*F* ^2^) = 0.103
*S* = 0.82
1853 reflections
248 parameters
H-atom parameters constrained
Δρ_max_ = 0.15 e Å^−3^
Δρ_min_ = −0.17 e Å^−3^

### 

Data collection: *CAD-4 EXPRESS* (Enraf–Nonius, 1994 ▶); cell refinement: *SET4* (de Boer & Duisenberg, 1984 ▶); data reduction: *MolEN* (Enraf–Nonius, 1990 ▶); program(s) used to solve structure: *SHELXS97* (Sheldrick, 2008 ▶); program(s) used to refine structure: *SHELXL97* (Sheldrick, 2008 ▶); molecular graphics: *XP* (Siemens, 1990 ▶); software used to prepare material for publication: *SHELXL97*.

## Supplementary Material

Crystal structure: contains datablocks global, I. DOI: 10.1107/S1600536808040397/nc2125sup1.cif

Structure factors: contains datablocks I. DOI: 10.1107/S1600536808040397/nc2125Isup2.hkl

Additional supplementary materials:  crystallographic information; 3D view; checkCIF report

## Figures and Tables

**Table 1:** Hydrogen-bond geometry (Å, °)

*D*—H⋯*A*	*D*—H	H⋯*A*	*D*⋯*A*	*D*—H⋯*A*
C5—H5⋯N1^i^	0.95	2.72	3.554 (4)	146

Symmetry code: (i) 
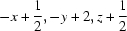
.

**Fig. 1.**
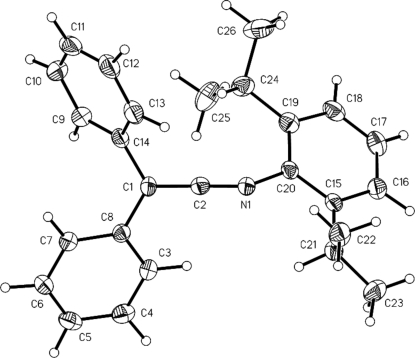
The molecular structure of the title compound, presenting the labelling scheme and 40% probability displacement ellipsoids for non-H atoms.

**Fig. 2.**
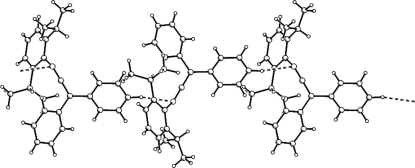
Infinite chains of the title compound realized by C—H···O hydrogen bonds.

## supplementary crystallographic information

### Comment

In the course of a study on the organometallic and catalytic chemistry of aromatic imines (Imhof, 1997*a*,*b*) we became interested in the reactivity of the vinylogous keteneimines. The latter are prepared by the elimination of water from the corresponding acetamides by P_2_O_5_ in anhydrous pyridine (Stevens & Singhal, 1964).

As expected the molecular structure of the title compound shows an almost linear cumulene system with an angle of 172.5 (4)° at the central C atom. The bonds C1—C2 and C2—N1 show bond lengths of 1.332 (5) and 1.213 (4) Å, respectively. The dihedral angle between the C1—C8—C14 plane and the aromatic substituent at the imine N atom measures to 52.4 (7)° which means that the substituents at the cumulene system do not show the expected orthogonal arrangement. This is most probably caused by the high steric requirements of the two isopropyl groups in *ortho*-position. A comparison with related aromatic diphenylvinylidene amines from the literature shows that the corresponding dihedral angle is close to 90° if there is no or just one *ortho*-substituent present in the aromatic group at nitrogen (*p*-Br-C_6_H_4_: 85.6°, Naqvi & Wheatley, 1970; *o*-Me-C_6_H_4_: 88.1°, Jochims *et al.*, 1984; *p*-(N═C═CPh_2_)-C_6_H_4_: 88.4°, Kuipers *et al.*, 1989; *p*-Me-C_6_H_4_: 83.9°, Naqvi & Wheatley, 1970). If both *ortho*-positions are substituted the conformation is no longer orthogonal (*o*-Me_2_-C_6_H_3_: 51.7°, Jochims *et al.*, 1984) as it is also observed for the title compound. The lone pair at nitrogen is involved in a weak intramolecular hydrogen bond (Desiraju & Steiner, 1999) interaction towards H5 leading to the formation of infinite chains.

### Experimental

The title compound was prepared following a literature method (Stevens & Singhal, 1964). A sample of 2 g (5.4 mmol) *N*-(2,6-Diisopropyl-phenyl)-2,2-diphenyl-acetamide was dissolved in 50 ml of anhydrous pyridine. To this solution 5 g P_2_O_5_ were added and the mixture was refluxed for 7 h. After cooling the solution was filtered and pyridine was evaporated resulting in a red oily residue. The oil was transferred to a short chromatography column and light petroleum (b.p. 40–60°C) was used to elute a yellow solution of the title compound. Concentration of the solution and cooling to 4°C led to the formation of crystalline material from which the crystal for the structure analysis described herein was collected (yield: 1.56 g, 82%). MS (EI) [m/*z*, %]: 353 (*M*^+^, 80), 338 (C_25_H_24_N^+^, 22), 186 (C_13_H_16_N^+^, 100), 165 (C_13_H_9_^+^, 50), 115 (C_9_H_7_^+^, 19), 91 (C_7_H_7_^+^, 37), 77 (C_6_H_5_^+^, 17), 55 (C_4_H_7_^+^, 11), 41 (C_3_H_5_^+^, 35). ^1^H NMR (CDCl_3_, 298 K) [p.p.m.]: 1.11 (12 H, d, ^3^J_HH_ = 6.8 Hz, CH_3_), 3.24 (2 H, h, ^3^J_HH_ = 6.8 Hz, CH), 7.05–7.34 (15 H, m, CH_ar_). ^13^C NMR (CDCl_3_, 298 K) [p.p.m.]: 22.4 (CH_3_), 28.5 (CH), 72.0 (═C), 123.4 (C_ar_H), 125.9 (C_ar_H), 126.3 (C_ar_H), 127.8 (C_ar_H), 128.7 (C_ar_H), 134.8 (C_ar_), 136.2 (C_ar_), 140.8 (C_ar_), 183.4 (═C═).

### Refinement

H atoms were positioned with idealized geometry at distances of 0.95 Å for aromatic C—H functions, 1.00 Å for aliphatic C—H bonds and 0.98 Å for methyl groups and were refined riding on their parent atoms with isotropic thermal parameters of 1.2 times the corresponding values of their parent atoms. In the absence of significant anomalous dispersion effects, Friedel pairs were averaged.

### Crystal data

**Table:**

C_26_H_27_N	*F*(000) = 760
*M**_r_* = 353.49	*D*_x_ = 1.141 Mg m^−^^3^
Orthorhombic, *P*2_1_2_1_2_1_	Mo *K*α radiation, λ = 0.71073 Å
Hall symbol: P 2ac 2ab	Cell parameters from 25 reflections
*a* = 8.082 (4) Å	θ = 20.9–35.5°
*b* = 14.308 (4) Å	µ = 0.07 mm^−^^1^
*c* = 17.790 (2) Å	*T* = 173 K
*V* = 2057 (1) Å^3^	Cube, pale yellow
*Z* = 4	0.3 × 0.2 × 0.2 mm

### Data collection

**Table:**

Enraf–Nonius CAD-4 diffractometer	*R*_int_ = 0.065
Radiation source: fine-focus sealed tube	θ_max_ = 24.0°, θ_min_ = 1.8°
graphite	*h* = −9→0
ω/2θ scans	*k* = −16→16
3554 measured reflections	*l* = 0→20
1853 independent reflections	3 standard reflections every 120 min
1531 reflections with *I* > 2σ(*I*)	intensity decay: <0.1%

### Refinement

**Table:**

Refinement on *F*^2^	Primary atom site location: structure-invariant direct methods
Least-squares matrix: full	Secondary atom site location: difference Fourier map
*R*[*F*^2^ > 2σ(*F*^2^)] = 0.040	Hydrogen site location: inferred from neighbouring sites
*wR*(*F*^2^) = 0.103	H-atom parameters constrained
*S* = 0.82	*w* = 1/[σ^2^(*F*_o_^2^) + (0.1319*P*)^2^ + 0.5946*P*] where *P* = (*F*_o_^2^ + 2*F*_c_^2^)/3
1853 reflections	(Δ/σ)_max_ < 0.001
248 parameters	Δρ_max_ = 0.15 e Å^−^^3^
0 restraints	Δρ_min_ = −0.17 e Å^−^^3^

### Special details

**Table:**

Geometry. All e.s.d.'s (except the e.s.d. in the dihedral angle between two l.s. planes) are estimated using the full covariance matrix. The cell e.s.d.'s are taken into account individually in the estimation of e.s.d.'s in distances, angles and torsion angles; correlations between e.s.d.'s in cell parameters are only used when they are defined by crystal symmetry. An approximate (isotropic) treatment of cell e.s.d.'s is used for estimating e.s.d.'s involving l.s. planes.
Refinement. Refinement on *F*^2^ for ALL reflections except for 9 with very negative *F*^2^ or flagged by the user for potential systematic errors. Weighted *R*-factors *wR* and all goodnesses of fit *S* are based on *F*^2^, conventional *R*-factors *R* are based on *F*, with *F* set to zero for negative *F*^2^. The observed criterion of *F*^2^ > σ(*F*^2^) is used only for calculating _R_factor_obs *etc*. and is not relevant to the choice of reflections for refinement. *R*-factors based on *F*^2^ are statistically about twice as large as those based on *F*, and *R*- factors based on ALL data will be even larger.

### Fractional atomic coordinates and isotropic or equivalent isotropic displacement parameters (Å^2^)

**Table:**

	*x*	*y*	*z*	*U*_iso_*/*U*_eq_	
N1	0.1809 (3)	0.90928 (16)	0.93459 (13)	0.0304 (6)	
C1	0.2195 (4)	1.06100 (19)	1.00668 (17)	0.0303 (7)	
C2	0.1892 (4)	0.98254 (19)	0.96930 (15)	0.0306 (7)	
C3	0.1148 (4)	0.9958 (2)	1.12860 (17)	0.0363 (7)	
H3	0.0626	0.9470	1.1012	0.044*	
C4	0.1008 (4)	0.9981 (2)	1.20605 (18)	0.0421 (8)	
H4	0.0401	0.9508	1.2314	0.051*	
C5	0.1749 (4)	1.0691 (2)	1.24668 (18)	0.0414 (8)	
H5	0.1636	1.0717	1.2998	0.050*	
C6	0.2652 (4)	1.1358 (2)	1.20919 (18)	0.0408 (8)	
H6	0.3178	1.1841	1.2370	0.049*	
C7	0.2810 (4)	1.1341 (2)	1.13188 (16)	0.0339 (7)	
H7	0.3444	1.1807	1.1071	0.041*	
C8	0.2041 (4)	1.06391 (18)	1.09007 (17)	0.0289 (6)	
C9	0.1828 (5)	1.2310 (2)	0.97473 (17)	0.0404 (8)	
H9	0.1050	1.2370	1.0144	0.049*	
C10	0.2210 (5)	1.3071 (2)	0.9299 (2)	0.0494 (9)	
H10	0.1695	1.3656	0.9395	0.059*	
C11	0.3325 (6)	1.2993 (3)	0.87148 (19)	0.0569 (11)	
H11	0.3577	1.3521	0.8411	0.068*	
C12	0.4071 (5)	1.2144 (3)	0.85749 (19)	0.0544 (10)	
H12	0.4830	1.2085	0.8170	0.065*	
C13	0.3720 (4)	1.1378 (2)	0.90200 (17)	0.0408 (8)	
H13	0.4248	1.0797	0.8923	0.049*	
C14	0.2595 (4)	1.1454 (2)	0.96113 (15)	0.0314 (6)	
C15	0.0304 (4)	0.7690 (2)	0.90761 (15)	0.0300 (7)	
C16	−0.1020 (4)	0.7236 (2)	0.87388 (17)	0.0374 (7)	
H16	−0.1141	0.6580	0.8796	0.045*	
C17	−0.2159 (4)	0.7728 (2)	0.83219 (19)	0.0432 (8)	
H17	−0.3071	0.7409	0.8103	0.052*	
C18	−0.1993 (4)	0.8682 (2)	0.82167 (17)	0.0406 (7)	
H18	−0.2781	0.9007	0.7918	0.049*	
C19	−0.0683 (4)	0.9176 (2)	0.85429 (16)	0.0335 (7)	
C20	0.0419 (4)	0.8665 (2)	0.89897 (14)	0.0282 (6)	
C21	0.1642 (4)	0.7162 (2)	0.95050 (17)	0.0364 (7)	
H21	0.1917	0.7540	0.9961	0.044*	
C22	0.3218 (5)	0.7091 (2)	0.9033 (2)	0.0461 (8)	
H22A	0.3554	0.7716	0.8869	0.055*	
H22B	0.3008	0.6698	0.8592	0.055*	
H22C	0.4103	0.6812	0.9336	0.055*	
C23	0.1115 (6)	0.6197 (2)	0.9776 (2)	0.0545 (10)	
H23A	0.1997	0.5926	1.0085	0.065*	
H23B	0.0906	0.5793	0.9342	0.065*	
H23C	0.0103	0.6252	1.0076	0.065*	
C24	−0.0519 (4)	1.0221 (2)	0.84131 (18)	0.0396 (8)	
H24	0.0658	1.0392	0.8521	0.048*	
C25	−0.1593 (5)	1.0765 (2)	0.8963 (3)	0.0586 (10)	
H25A	−0.1315	1.0581	0.9478	0.070*	
H25B	−0.2761	1.0628	0.8865	0.070*	
H25C	−0.1394	1.1436	0.8899	0.070*	
C26	−0.0872 (5)	1.0502 (3)	0.7602 (2)	0.0623 (11)	
H26A	−0.2043	1.0392	0.7488	0.075*	
H26B	−0.0183	1.0129	0.7262	0.075*	
H26C	−0.0618	1.1167	0.7534	0.075*	

### Atomic displacement parameters (Å^2^)

**Table:**

	*U*^11^	*U*^22^	*U*^33^	*U*^12^	*U*^13^	*U*^23^
N1	0.0354 (14)	0.0255 (12)	0.0303 (12)	0.0015 (11)	−0.0040 (11)	−0.0019 (10)
C1	0.0340 (16)	0.0248 (14)	0.0322 (14)	−0.0013 (14)	−0.0016 (13)	−0.0040 (12)
C2	0.0334 (15)	0.0293 (15)	0.0291 (13)	0.0027 (13)	−0.0018 (13)	0.0026 (13)
C3	0.0431 (17)	0.0271 (13)	0.0387 (15)	−0.0057 (15)	−0.0027 (15)	−0.0019 (13)
C4	0.0489 (18)	0.0383 (16)	0.0391 (16)	−0.0007 (17)	0.0021 (16)	0.0087 (14)
C5	0.0473 (19)	0.0491 (18)	0.0279 (14)	0.0042 (17)	−0.0023 (15)	0.0052 (14)
C6	0.0512 (18)	0.0367 (16)	0.0347 (15)	−0.0019 (17)	−0.0147 (15)	−0.0037 (13)
C7	0.0391 (16)	0.0298 (14)	0.0329 (15)	−0.0042 (15)	−0.0033 (13)	0.0013 (12)
C8	0.0297 (14)	0.0254 (13)	0.0315 (14)	0.0021 (13)	−0.0019 (13)	−0.0005 (11)
C9	0.0502 (19)	0.0352 (16)	0.0358 (16)	−0.0024 (16)	−0.0036 (16)	0.0020 (13)
C10	0.065 (2)	0.0340 (16)	0.0491 (19)	−0.0055 (18)	−0.0197 (19)	0.0057 (15)
C11	0.079 (3)	0.059 (2)	0.0328 (17)	−0.040 (2)	−0.0121 (19)	0.0129 (17)
C12	0.067 (2)	0.063 (2)	0.0333 (17)	−0.031 (2)	−0.0003 (18)	−0.0005 (16)
C13	0.0444 (18)	0.0420 (16)	0.0360 (15)	−0.0120 (16)	0.0041 (14)	−0.0062 (15)
C14	0.0365 (15)	0.0316 (14)	0.0260 (13)	−0.0075 (15)	−0.0038 (12)	−0.0018 (12)
C15	0.0369 (16)	0.0290 (14)	0.0242 (13)	0.0021 (13)	−0.0013 (14)	−0.0039 (12)
C16	0.0438 (18)	0.0333 (15)	0.0351 (15)	−0.0013 (15)	−0.0010 (15)	−0.0049 (14)
C17	0.0385 (18)	0.0483 (18)	0.0427 (17)	−0.0083 (16)	−0.0062 (16)	−0.0083 (16)
C18	0.0399 (17)	0.0486 (18)	0.0334 (15)	0.0025 (17)	−0.0105 (14)	−0.0015 (14)
C19	0.0338 (16)	0.0365 (16)	0.0303 (14)	0.0024 (14)	0.0029 (13)	−0.0040 (13)
C20	0.0319 (14)	0.0284 (13)	0.0242 (13)	0.0035 (13)	0.0007 (12)	−0.0048 (11)
C21	0.0454 (18)	0.0283 (14)	0.0354 (15)	0.0048 (14)	−0.0075 (15)	−0.0029 (13)
C22	0.0431 (18)	0.0409 (17)	0.054 (2)	0.0099 (16)	−0.0056 (17)	−0.0066 (16)
C23	0.065 (2)	0.0357 (17)	0.063 (2)	0.0021 (19)	−0.015 (2)	0.0084 (17)
C24	0.0386 (17)	0.0363 (16)	0.0438 (17)	0.0075 (15)	−0.0028 (15)	0.0061 (14)
C25	0.055 (2)	0.0350 (17)	0.086 (3)	0.0103 (18)	0.011 (2)	−0.0023 (19)
C26	0.060 (2)	0.064 (2)	0.064 (2)	0.000 (2)	−0.010 (2)	0.030 (2)

### Geometric parameters (Å, °)

**Table:**

N1—C2	1.218 (4)	C15—C20	1.406 (4)
N1—C20	1.428 (4)	C15—C21	1.523 (4)
C1—C2	1.328 (4)	C16—C17	1.376 (5)
C1—C14	1.490 (4)	C16—H16	0.9500
C1—C8	1.489 (4)	C17—C18	1.385 (5)
C3—C8	1.393 (4)	C17—H17	0.9500
C3—C4	1.383 (4)	C18—C19	1.399 (5)
C3—H3	0.9500	C18—H18	0.9500
C4—C5	1.384 (5)	C19—C20	1.400 (4)
C4—H4	0.9500	C19—C24	1.519 (4)
C5—C6	1.374 (5)	C21—C23	1.523 (5)
C5—H5	0.9500	C21—C22	1.529 (5)
C6—C7	1.381 (4)	C21—H21	1.0000
C6—H6	0.9500	C22—H22A	0.9800
C7—C8	1.395 (4)	C22—H22B	0.9800
C7—H7	0.9500	C22—H22C	0.9800
C9—C10	1.385 (5)	C23—H23A	0.9800
C9—C14	1.393 (5)	C23—H23B	0.9800
C9—H9	0.9500	C23—H23C	0.9800
C10—C11	1.380 (6)	C24—C26	1.525 (5)
C10—H10	0.9500	C24—C25	1.521 (5)
C11—C12	1.379 (6)	C24—H24	1.0000
C11—H11	0.9500	C25—H25A	0.9800
C12—C13	1.381 (5)	C25—H25B	0.9800
C12—H12	0.9500	C25—H25C	0.9800
C13—C14	1.395 (4)	C26—H26A	0.9800
C13—H13	0.9500	C26—H26B	0.9800
C15—C16	1.388 (5)	C26—H26C	0.9800
			
C2—N1—C20	129.6 (3)	C16—C17—H17	119.6
C2—C1—C14	116.9 (3)	C18—C17—H17	119.6
C2—C1—C8	120.5 (3)	C17—C18—C19	121.0 (3)
C14—C1—C8	122.5 (2)	C17—C18—H18	119.5
N1—C2—C1	172.5 (3)	C19—C18—H18	119.5
C8—C3—C4	121.1 (3)	C18—C19—C20	117.0 (3)
C8—C3—H3	119.5	C18—C19—C24	120.0 (3)
C4—C3—H3	119.5	C20—C19—C24	123.0 (3)
C5—C4—C3	120.2 (3)	C15—C20—C19	122.6 (3)
C5—C4—H4	119.9	C15—C20—N1	115.4 (2)
C3—C4—H4	119.9	C19—C20—N1	121.9 (3)
C6—C5—C4	119.1 (3)	C23—C21—C15	114.2 (3)
C6—C5—H5	120.4	C23—C21—C22	110.3 (3)
C4—C5—H5	120.4	C15—C21—C22	110.4 (3)
C5—C6—C7	121.3 (3)	C23—C21—H21	107.2
C5—C6—H6	119.4	C15—C21—H21	107.2
C7—C6—H6	119.4	C22—C21—H21	107.2
C6—C7—C8	120.2 (3)	C21—C22—H22A	109.5
C6—C7—H7	119.9	C21—C22—H22B	109.5
C8—C7—H7	119.9	H22A—C22—H22B	109.5
C3—C8—C7	118.1 (3)	C21—C22—H22C	109.5
C3—C8—C1	120.9 (3)	H22A—C22—H22C	109.5
C7—C8—C1	120.9 (3)	H22B—C22—H22C	109.5
C10—C9—C14	119.5 (3)	C21—C23—H23A	109.5
C10—C9—H9	120.3	C21—C23—H23B	109.5
C14—C9—H9	120.3	H23A—C23—H23B	109.5
C11—C10—C9	121.0 (3)	C21—C23—H23C	109.5
C11—C10—H10	119.5	H23A—C23—H23C	109.5
C9—C10—H10	119.5	H23B—C23—H23C	109.5
C12—C11—C10	119.5 (3)	C19—C24—C26	112.8 (3)
C12—C11—H11	120.2	C19—C24—C25	110.8 (3)
C10—C11—H11	120.2	C26—C24—C25	111.5 (3)
C13—C12—C11	120.4 (3)	C19—C24—H24	107.1
C13—C12—H12	119.8	C26—C24—H24	107.1
C11—C12—H12	119.8	C25—C24—H24	107.1
C12—C13—C14	120.3 (3)	C24—C25—H25A	109.5
C12—C13—H13	119.9	C24—C25—H25B	109.5
C14—C13—H13	119.9	H25A—C25—H25B	109.5
C9—C14—C13	119.3 (3)	C24—C25—H25C	109.5
C9—C14—C1	121.4 (3)	H25A—C25—H25C	109.5
C13—C14—C1	119.2 (3)	H25B—C25—H25C	109.5
C16—C15—C20	117.8 (3)	C24—C26—H26A	109.5
C16—C15—C21	122.1 (3)	C24—C26—H26B	109.5
C20—C15—C21	120.0 (3)	H26A—C26—H26B	109.5
C17—C16—C15	120.7 (3)	C24—C26—H26C	109.5
C17—C16—H16	119.7	H26A—C26—H26C	109.5
C15—C16—H16	119.7	H26B—C26—H26C	109.5
C16—C17—C18	120.8 (3)		
			
C20—N1—C2—C1	−169 (2)	C8—C1—C14—C13	137.9 (3)
C14—C1—C2—N1	82 (2)	C20—C15—C16—C17	1.3 (4)
C8—C1—C2—N1	−101 (2)	C21—C15—C16—C17	−176.5 (3)
C8—C3—C4—C5	0.5 (5)	C15—C16—C17—C18	1.3 (5)
C3—C4—C5—C6	−1.4 (5)	C16—C17—C18—C19	−1.3 (5)
C4—C5—C6—C7	1.0 (5)	C17—C18—C19—C20	−1.3 (5)
C5—C6—C7—C8	0.3 (5)	C17—C18—C19—C24	179.4 (3)
C4—C3—C8—C7	0.8 (5)	C16—C15—C20—C19	−4.1 (4)
C4—C3—C8—C1	179.7 (3)	C21—C15—C20—C19	173.8 (3)
C6—C7—C8—C3	−1.2 (4)	C16—C15—C20—N1	−179.8 (2)
C6—C7—C8—C1	179.9 (3)	C21—C15—C20—N1	−1.9 (4)
C2—C1—C8—C3	−18.4 (5)	C18—C19—C20—C15	4.0 (4)
C14—C1—C8—C3	158.0 (3)	C24—C19—C20—C15	−176.7 (3)
C2—C1—C8—C7	160.5 (3)	C18—C19—C20—N1	179.5 (3)
C14—C1—C8—C7	−23.1 (4)	C24—C19—C20—N1	−1.3 (4)
C14—C9—C10—C11	0.7 (5)	C2—N1—C20—C15	−139.4 (3)
C9—C10—C11—C12	0.1 (5)	C2—N1—C20—C19	44.9 (4)
C10—C11—C12—C13	−0.7 (6)	C16—C15—C21—C23	−21.2 (4)
C11—C12—C13—C14	0.6 (5)	C20—C15—C21—C23	161.0 (3)
C10—C9—C14—C13	−0.7 (5)	C16—C15—C21—C22	103.7 (3)
C10—C9—C14—C1	−178.5 (3)	C20—C15—C21—C22	−74.0 (3)
C12—C13—C14—C9	0.1 (5)	C18—C19—C24—C26	−41.8 (4)
C12—C13—C14—C1	178.0 (3)	C20—C19—C24—C26	139.0 (3)
C2—C1—C14—C9	132.2 (3)	C18—C19—C24—C25	84.0 (4)
C8—C1—C14—C9	−44.3 (4)	C20—C19—C24—C25	−95.2 (4)
C2—C1—C14—C13	−45.6 (4)		

### Hydrogen-bond geometry (Å, °)

**Table:**

*D*—H···*A*	*D*—H	H···*A*	*D*···*A*	*D*—H···*A*
C5—H5···N1^i^	0.95	2.72	3.554 (4)	146

Symmetry codes: (i) −*x*+1/2, −*y*+2, *z*+1/2.

## References

[bb1] Boer, J. L. de & Duisenberg, A. J. M. (1984). *Acta Cryst.* A**40**, C410.

[bb2] Desiraju, G. R. & Steiner, T. (1999). *The Weak Hydrogen Bond*, pp. 29–121. Oxford University Press.

[bb3] Enraf–Nonius (1990). *MolEN* Enraf–Nonius, Delft, The Netherlands.

[bb4] Enraf–Nonius (1994). *CAD-4 EXPRESS* Enraf–Nonius, Delft, The Netherlands.

[bb5] Imhof, W. (1997*a*). *J. Organomet. Chem.* **533**, 31–43.

[bb6] Imhof, W. (1997*b*). *J. Organomet. Chem.* **541**, 109–116.

[bb7] Jochims, J. C., Lambrecht, J., Burkert, U., Zsolnai, L. & Huttner, G. (1984). *Tetrahedron*, **40**, 893–903.

[bb8] Kuipers, W., Kanters, J. A. & Schouten, A. (1989). *Acta Cryst.* C**45**, 482–485.

[bb9] Naqvi, R. R. & Wheatley, P. J. (1970). *J. Chem. Soc. A*, pp. 2053–2058.

[bb10] Sheldrick, G. M. (2008). *Acta Cryst.* A**64**, 112–122. 10.1107/S0108767307043930 18156677

[bb11] Siemens (1990). *XP* Siemens Analytical X-ray Instruments Inc., Madison, Wisconsin, USA.

[bb12] Stevens, C. L. & Singhal, G. H. (1964). *J. Org. Chem.* **29**, 34–37.

